# Clinical and genetic interpretation of uncertain *DMD* missense variants: evidence from mRNA and protein studies

**DOI:** 10.1186/s13023-024-03128-7

**Published:** 2024-03-14

**Authors:** Zhiying Xie, Chang Liu, Haiyan Yu, Zhihao Xie, Chengyue Sun, Ying Zhu, Xiaoyu Hu, Li Bai, Luhua Wei, Peng Sun, Yanyu Lu, Yunlong Lu, Yawen Zhao, Wei Zhang, Zhaoxia Wang, Lingchao Meng, Yun Yuan

**Affiliations:** 1 Department of Neurology, Peking University First Hospital, No. 8 Xishiku Street, Xicheng District, Beijing, 100034 China; 2 Department of Clinical Laboratory, Peking University First Hospital, Beijing, 100034 China; 3 Department of Epidemiology and Biostatistics, West China School of Public Health and West China Fourth Hospital, Sichuan University, Chengdu, 610041 China; 4 Department of Neurology, Peking University People’s Hospital, Beijing, 100044 China; 5 Department of Radiology, Peking University First Hospital, Beijing, 100034 China

**Keywords:** Dystrophinopathies, *DMD*, Missense variants, Aberrant splicing

## Abstract

**Background:**

Pathogenic missense variants in the *dystrophin* (*DMD*) gene are rarely reported in dystrophinopathies. Most *DMD* missense variants are of uncertain significance and their pathogenicity interpretation remains complicated. We aimed to investigate whether *DMD* missense variants would cause aberrant splicing and re-interpret their pathogenicity based on mRNA and protein studies.

**Methods:**

Nine unrelated patients who had an elevated serum creatine kinase level with or without muscle weakness were enrolled. They underwent a detailed clinical, imaging, and pathological assessment. Routine genetic testing and muscle-derived mRNA and protein studies of *dystrophin* and *sarcoglycan* genes were performed in them.

**Results:**

Three of the 9 patients presented with a Duchenne muscular dystrophy (DMD) phenotype and the remaining 6 patients had a suspected diagnosis of Becker muscular dystrophy (BMD) or sarcoglycanopathy based on their clinical and pathological characteristics. Routine genetic testing detected only 9 predicted *DMD* missense variants in them, of which 6 were novel and interpreted as uncertain significance. Muscle-derived mRNA studies of *sarcoglycan* genes didn’t reveal any aberrant transcripts in them. *Dystrophin* mRNA studies confirmed that 3 predicted *DMD* missense variants (c.2380G > C, c.4977C > G, and c.5444A > G) were in fact splicing and frameshift variants due to aberrant splicing. The 9 *DMD* variants were re-interpreted as pathogenic or likely pathogenic based on mRNA and protein studies. Therefore, 3 patients with *DMD* splicing variants and 6 patients with confirmed *DMD* missense variants were diagnosed with DMD and BMD, respectively.

**Conclusion:**

Our study highlights the importance of muscle biopsy and aberrant splicing for clinical and genetic interpretation of uncertain *DMD* missense variants.

## Introduction

An accurate genetic diagnosis of monogenic diseases is crucial for disease management and family genetic counselling. Dystrophinopathies, a collective term for Duchenne muscular dystrophy (DMD), Becker muscular dystrophy (BMD), and X-linked dilated cardiomyopathy, are caused by pathogenic variants in the dystrophin-encoding *DMD* gene [1]. Dystrophinopathies primarily affect male patients as the *DMD* gene is located on the X-chromosome. DMD patients have a severe phenotype and are caused by *DMD* variants that completely abolish dystrophin function in myofibers [1, 2], including frameshift and nonsense variants. BMD patients typically present a milder phenotype and have inframe *DMD* variants that maintain the open reading-frame, allowing the production of semi-functional dystrophin in skeletal muscles [2, 3]. The genetic spectrum of dystrophinopathies consists of both coding and non-coding *DMD* variants, ranging from single nucleotide variants (SNVs) to complex structural variants [2, 4]. There are abundant repetitive elements and common fragile sites in *DMD* introns that can mediate large genomic rearrangements [5]. Therefore, large genomic rearrangements including exonic deletions/duplications (copy number variants) and structural variants are quite common in *DMD*, accounting for about 80% of all pathogenic *DMD* variants [6, 7]. The remaining about 20% are primarily small pathogenic variants, including small deletions, small insertions, small deletion-insertions, nonsense, splicing, and missense variants [6, 7].

Most missense variants in *DMD* are of uncertain significance and their pathogenicity interpretation remains difficult and complicated. Pathogenic *DMD* missense variants are rarely (less than 1%) reported in dystrophinopathies [6]. Most previously reported pathogenic missense variants in *DMD* are located in key functional domains of dystrophin protein, including the N-terminal actin binding domain (ABD1) which are commonly associated with a BMD phenotype [8] and the conserved ZZ domain of the C-terminal region which usually cause a severe DMD phenotype [9]. However, the effects of missense variants on *DMD* pre-mRNA splicing and dystrophin expression is often unavailable. Aberrant splicing events induced by missense variants have been observed in some other monogenic disease genes [10–14], but only in one DMD patient with c.5444A > G in *DMD* [15]. The possibility that predicted *DMD* missense variants may in fact be splicing and frameshift variants due to aberrant splicing and thus lead to a severe DMD phenotype should be considered. *DMD* missense variants may change the essential splicing signals and/or splicing regulatory elements and cause aberrant splicing of *DMD* pre-mRNA. This requires the support of mRNA and protein studies and is especially important when interpreting missense variants’ pathogenicity and their phenotypic contributions. In this study, we aimed to investigate whether *DMD* missense variants would cause aberrant splicing and re-interpret their pathogenicity based on evidence derived from clinical, bioinformatic, mRNA, and protein studies.

## Methods

### Patients

Nine patients from 9 unrelated families who had an elevated serum creatine kinase (CK) level with or without muscle weakness were enrolled in this study, as an elevated serum CK level and muscle weakness indicated a skeletal muscle disease. Clinical characteristics were ascertained by review of their medical records and a detailed neurological examination. Muscle strength of each muscle group was graded by manual muscle testing.

### Muscle MRI examinations

Muscle magnetic resonance imaging (MRI) examinations of the pelvis and thigh muscles were performed in the 9 enrolled patients according to a previous study [16]. Fatty infiltration of each individual muscle was graded and scored on axial T1-weighted images according to a modified 0–5-point Mercuri’s scale [17] as follows: score 0 (stage 0), normal appearance with no muscle fatty infiltration; score 1 (stage 1), a few scattered areas of T1 hyperintensity; score 2 (stage 2a), numerous discrete areas of T1 hyperintensity with less than 30% of the individual muscle volume; score 3 (stage 2b), increased areas of confluent T1 hyperintensity with 30–60% of the individual muscle volume; score 4 (stage 3), confluent areas of T1 hyperintensity with more than 60% of the individual muscle volume; and score 5 (stage 4), complete muscle fatty infiltration with entirely confluent T1 hyperintensity. Scores 0 and 1 indicate mild, scores 2 and 3 indicate moderate, and scores 4 and 5 indicate severe fatty infiltration of the individual muscle. All scans were scored by two independent observers who were blinded to the clinical and genetic information; two observers interpreted independently and reached consensus after discussion.

### Routine genetic testing

On the basis of their hyperCKemia with or without muscle weakness phenotype, we performed multiplex ligation-dependent probe amplification-analysis of *SGCA*, *SGCB*, *SGCD*, *SGCG*, and *DMD* [18] in the 9 enrolled patients to detect possible disease-causing exonic deletions and/or duplications. To detect pathogenic SNVs, small deletions, small insertions, and small deletion-insertions, a targeted next-generation sequencing (NGS) panel [4] was performed in them, which covered exonic regions and flanking intronic sequences of protein-coding genes known to be associated with genetic neuromuscular diseases. Genomic Sanger sequencing was performed to validate the DNA variants detected by the targeted NGS panel.

### Muscle-derived protein and mRNA studies

Muscle biopsies were performed in the 9 enrolled patients and a healthy control subject. Muscle biopsy specimens were rapidly frozen in isopentane, cooled in liquid nitrogen, and then stored at − 80 °C. Routine histochemical, histological, and immunohistochemical staining were performed in the 9 patients according to previous protocols [4]. Primary monoclonal antibodies against the different domains of dystrophin and sarcoglycan proteins were used, including dystrophin-N (amino-terminal; DYS3), dystrophin-C (carboxyl-terminal; DYS2), dystrophin-R (rod-domain; DYS1), α-sarcoglycan, β-sarcoglycan, and γ-sarcoglycan (Novocastra Laboratories, Newcastle) [4].

Total muscle RNA was extracted from the remaining muscle biopsy specimens using Trizol (Invitrogen, La Jolla, CA) and subsequently retrotranscribed to cDNA using a HiScript II Q RT SuperMix kit (Vazyme, Nanjing, China) according to the manufacturers’ recommended protocols. Full length cDNA of *DMD* (NM_004006.2), *SGCG* (NM_000231.2), *SGCD* (NM_000337.5), *SGCB* (NM_000232.4), and *SGCA* (NM_000023.2) were respectively amplified and Sanger sequenced in different overlapping cDNA fragments using a set of primers as described in a previous study [18]. Aberrant cDNA fragments were analyzed by gel electrophoresis. In addition, normal and blank controls were included in each gel electrophoresis analysis. If the Sanger sequencing trace for an aberrant cDNA fragment appeared to contain multiple sequences, the purified PCR product for that amplicon was TA cloned (TA cloning [18]), and individual clones were analyzed and sequenced, allowing identification of all transcripts.

### In silico splicing prediction

The Maximum Entropy Scan (MaxEntScan) [19], Human Splicing Finder (HSF) [20], and SpliceAI [21] were used to predict whether detected genomic variants would cause potential splicing alterations. The MaxEntScan tool assigns a log odds ratio to donor or acceptor splice site (5′ ss or 3′ ss) sequences; higher scores indicate a greater probability that the input sequence could be used as a splice site. The margins for HSF are between 0 to100 and higher scores indicate a greater potential for a splice site. SpliceAI estimates splicing alterations caused by SNVs and calculates the probability as delta scores. Higher delta scores indicate a greater probability that the SNV would cause aberrant splicing.

### Pathogenicity interpretation of detected variants

The genomic variants, RNA variants, and protein variants detected in our study were described according to the Human Genome Variation Society nomenclature [22]. Pathogenicity of each detected genomic variant was interpreted and classified according to the American College of Medical Genetics and Genomics and Association for Molecular Pathology (ACMG-AMP) guidelines [23]. Each detected genomic variant was interpreted as a pathogenic, likely pathogenic, uncertain significance, likely benign, or benign variant according to the rules specified in the ACMG-AMP guidelines [23].

When assessing the frequencies of detected variants in large populations, the gnomAD was screened. The evidence for pathogenicity was deemed to be moderate (PM2) for detected variants that were absent or present at extremely low frequencies with minor allele frequency less than 0.01% in gnomAD. Multiple pieces of computational evidence were derived from different in silico predictions where the PolyPhen-2, Mutation Taster, SIFT, and CADD were used to predict deleteriousness and the GERP was used to assess evolutionary conservation [23]. We used the wInterVar tool [24] to automatically generate predictions on 6 (PS1, PM1, PM5, PP2, BP1, and BP7) of 28 criteria specified in the ACMG-AMP guidelines; the rest were interpreted by manual review and adjustment based on variants’ detailed information and our own domain knowledge. Then, these criteria were combined to arrive at a final classification.

## Results

### Clinical and pathological characteristics

**Fig. 1:**
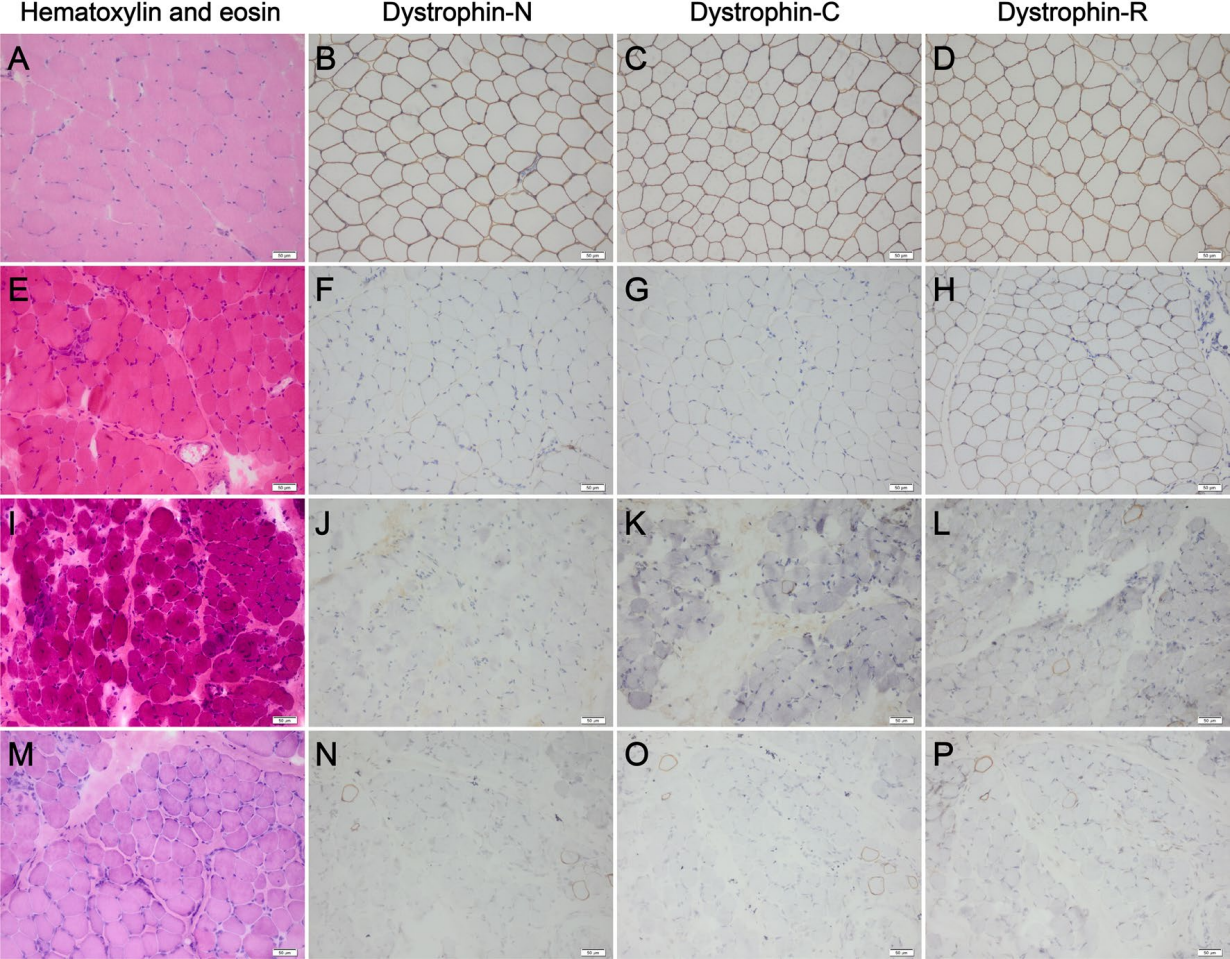
Pathologic changes and immunohistochemical staining of dystrophin in our enrolled patients. **A** Hematoxylin and eosin staining showing no pathologic changes. **E**, **I**, and **M** Hematoxylin and eosin staining showing a muscular dystrophic pattern. **B**–**D** A normal control showing positive dystrophin expression. **F**–**H** Patient 1 showing a severe reduction of dystrophin-N and dystrophin-C and a mild to partial reduction of dystrophin-R. **J**–**L** Patient 2 showing absence of dystrophin-N, absence with isolated revertant fibers of dystrophin-C, and absence with isolated revertant fibers and traces of dystrophin-R. **N**–**P** Patient 3 showing absence with a few revertant fibers of dystrophin-N, dystrophin-C, and dystrophin-R. **A**–**D** normal control; **E**–**H** patient 1; **I**–**L** patient 2; **M**–**P** patient 3

The detailed clinical and pathological characteristics of the 9 patients enrolled in this study were summarized in Table 1. Three of the 9 patients (P1–P3) presented with a DMD phenotype. Patient 1 is a 4.3-year-old boy who presented to our hospital because of a finding of hyperCKemia (7621–14,781 IU/L; normal 25–195 IU/L) and fatigue since 3.8-years of age. Physical examination confirmed that he had proximal muscle weakness, positive Gowers' sign, and calf hypertrophy. Patient 2 is an 8.8-year-old boy. He presented to our hospital because of delayed motor milestones and an elevated serum CK level (13,272 IU/L). He had a positive Gowers’ sign at 3-years of age and waddling gait at 8-years of age. Currently, he has moderate proximal muscle weakness, calf hypertrophy, and bilateral tendon contractures. Patient 3 is a 7-year-old boy who had difficulties in running and jumping around 3-years of age. He presented a positive Gowers’ sign at 4-years of age and waddling gait at 6.5-years of age. He now has calf hypertrophy, bilateral tendon contractures, and severe muscle weakness confirmed by physical examination. His serum CK level was markedly elevated with a value of 25,440 IU/L. All the 3 patients showed a muscular dystrophic pattern and complete deficiency or severe reduction of dystrophin-C and dystrophin-N, and variable reduction of dystrophin-R ranging from absence to a mild reduction (Fig. 1). In addition, immunohistochemical staining also revealed variable reduction of α-, β-, and γ-sarcoglycan in the 3 patients. However, the reduction of sarcoglycans was less severe than the reduction of dystrophin, indicating that the reduction of sarcoglycans was secondary changes.

**Table 1:** Clinical and pathological features of the nine enrolled patients

Patient number	Phenotype	Age, years/sex	Age at onset, years	Symptom(s) at onset	Calf hypertrophy	Tendon contractures	Muscle pain	Age at appearance of Gowers' sign, years	Age at waddling gait, years
P1	DMD	4.3	3.8	HyperCKemia; fatigue	+	–	–	4.3	–
P2	DMD	8.8	3	Delayed motor milestones	+	+	–	3	8
P3	DMD	7	3.5	Difficulties in running and jumping	+	+	–	4	6.5
P4	BMD	4.1	3	Difficulties in running	+	–	–	–	–
P5	BMD	17.2	12	Progressive lower limb weakness	+	–	–	12	–
P6	BMD	3.9	3.3	HyperCKemia	+	–	–	–	–
P7	BMD	4.7	2	Myalgia	–	–	+	–	–
P8	BMD	7.5	5.7	HyperCKemia	–	–	–	–	–
P9	BMD	3.6	3	Myalgia	–	–	+	–	–

Patient number	Age at loss of ambulation, years	Distribution of weakness	CK (IU/L)	Pathological pattern	Dystrophin-N	Dystrophin-C	Dystrophin-R	α-sarcoglycan	β-sarcoglycan	γ-sarcoglycan
P1	–	Proximal	7621	Dystrophic	+ /–	+ /–	+ ~ + +	+ + +	+ + ~ + + +	+ + +
P2	–	Proximal	13,272	Dystrophic	–	–	– ~ + /–	+ + +	+ + ~ + + +	+ + ~ + + +
P3	–	Generalized	25,440	Dystrophic	–	–	–	+ /–	+ /–	+ + +
P4	–	None	1544	Dystrophic	+ +	+ +	+ +	+ + +	+ +	+ + ~ + + +
P5	–	Proximal	7655	Dystrophic	– ~ + /–	+ +	+ + +	+ + +	+ + +	+ + +
P6	–	None	6223	Dystrophic	+ /–	+	+ +	+ + ~ + + +	+ /– ~ +	+ + +
P7	–	None	1474	Myopathic changes	+ +	+ + +	+ + +	+ + +	+ +	+ + +
P8	–	None	3832	Myopathic changes	+ +	+ +	+ +	+ + ~ + + +	+ + ~ + + +	+ + ~ + + +
P9	–	None	1807	Mild myopathic changes	+ +	+ +	+ + +	+ + +	+ +	+ + +

Protein expression based on immunohistochemical staining sections was graded into absence with or without isolated revertant fibers –, severe reduction + /–, partial reduction + , slight reduction +  + , and positive expression +  +  + . Phenotype of a patient with dystrophinopathy was determined as BMD or DMD according to the following criteria [4]: BMD, presenting with no obvious muscle weakness by 5 years of age and a slight to partial reduction in the expression of dystrophin-C regardless of the expression of dystrophin-R and dystrophin-N; DMD, proximal muscle weakness evident by 5 years of age and complete or almost complete deficiency of dystrophin, especially the dystrophin-C. DMD, Duchenne muscular dystrophy; BMD, Becker muscular dystrophy; CK, creatine kinase

Six of the 9 enrolled patients (P4–P9) had a suspected diagnosis of BMD or sarcoglycanopathy based on their clinical and pathological characteristics. The median age of the 6 patients was 4.4 (range 3.6–17.2) years. The median onset age was 3.2 (range 2.0–12.0) years and the median disease duration was 1.5 (range 0.5–5.2) years. Two patients (P6 and P8) were enrolled because of a finding of hyperCKemia. The onset symptoms were associated with proximal lower limb weakness in 2 patients (P4 and P5). The onset symptom of the remaining 2 patients (P7 and P9) was myalgia. Motor signs included calf hypertrophy (3/6), muscle pain (2/6), and positive Gowers’ sign (1/6). All the 6 patients are still independently ambulant. Five of the 6 patients (except patient 5) had no obvious muscle weakness confirmed by physical examination. Serum CK level was elevated in all 6 patients (range 1474–7655 IU/L). Muscle biopsies revealed that 3 patients (P4–P6) had muscular dystrophic changes and 3 patients (P7–P9) had myopathic changes. All 6 patients showed demonstrable expression of dystrophin-C, a slight to severe reduction of dystrophin-N, and a slight reduction to normal expression of dystrophin-R. The 6 patients also showed a slight reduction to normal expression of α-sarcoglycan and γ-sarcoglycan, and a partial reduction to normal expression of β-sarcoglycan. It could not be determined which one was the primarily affected among dystrophin, α-sarcoglycan, β-sarcoglycan, and γ-sarcoglycan, as there was a similar reduction of dystrophin and sarcoglycans in the 6 patients. Therefore, they were suspected of having a molecular diagnosis of BMD or sarcoglycanopathy.

### Muscle MRI features

**Fig. 2:**
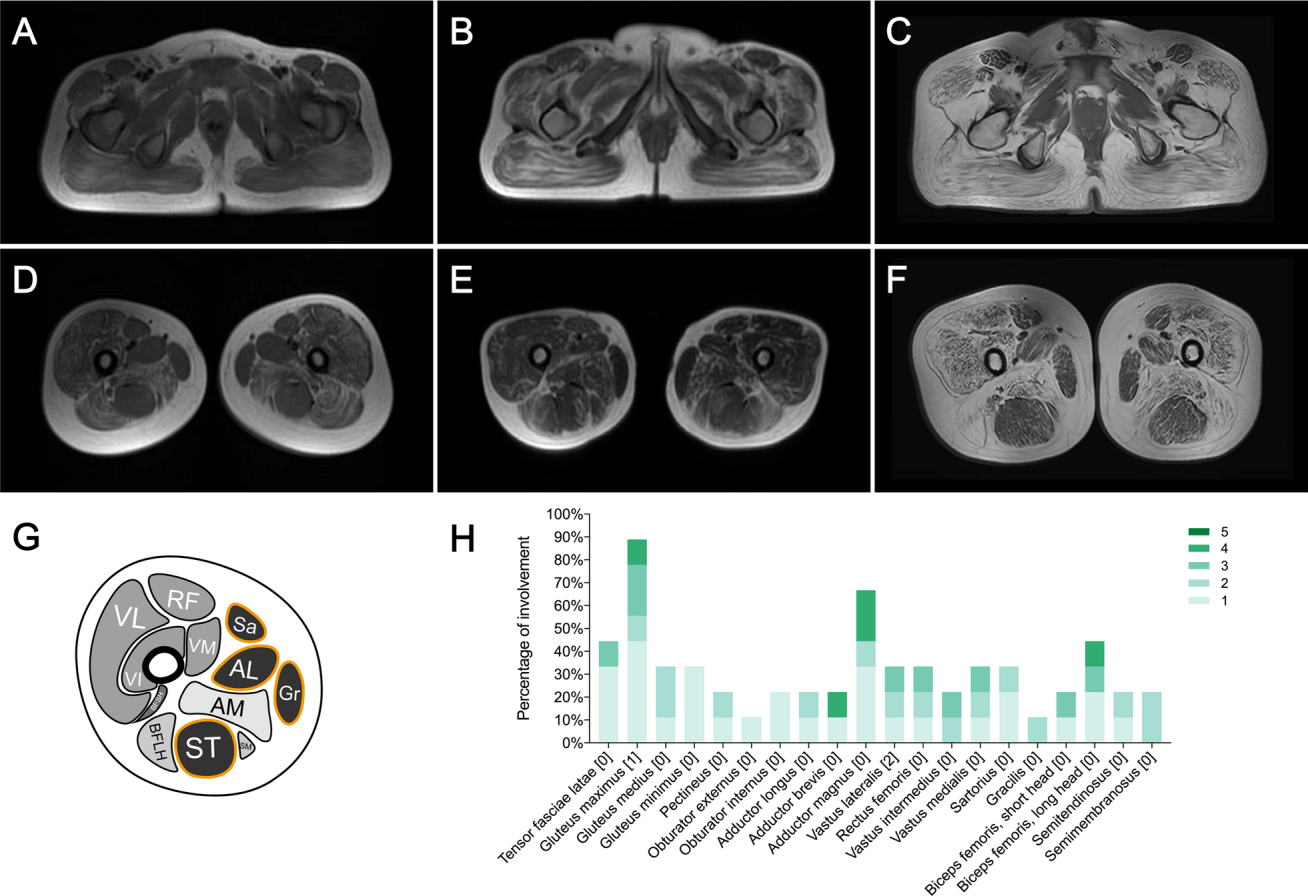
Muscle imaging studies of the nine enrolled patients. **A**–**C** Examples of muscle fatty infiltration at the pelvis level. **D**–**F** Examples of muscle fatty infiltration showing the trefoil with single fruit sign at the proximal thigh level, consisting of three leaflets formed by relative sparing of the sartorius, adductor longus, and gracilis muscles and one single fruit formed by relative sparing of the semitendinosus muscle **G**. **H** Frequency of fatty infiltration of each individual muscle was represented as a percentage of all patients. Green bars representing the percentage of each muscle affected for each score. The numbers within the square brackets indicating the median score for each individual muscle. **A** and **D** patient 2; **B** and **E** patient 3; **C** and **F** patient 5

The overall distribution and extent of muscle fatty infiltration of affected thigh and pelvis muscles were bilaterally symmetrical on axial T1-weighted images (Fig. 2A–F). Percentages of the extent of muscle fatty infiltration for each individual muscle were summarized in Fig. 2H. At the pelvis level, the gluteus maximus muscle was the most affected, with 88.89% showing muscle fatty infiltration, followed by the tensor fasciae latae (44.44), gluteus medius (33.33%), and gluteus minimus muscles (33.33%). The pectineus, obturator internus, and obturator externus muscles were relatively spared. At the thigh level, the adductor magnus muscle was the most affected, with 33.33% showing moderate or severe fatty infiltration. The rectus femoris, vastus lateralis, vastus intermedius, vastus medialis, semimembranosus, and long head of biceps femoris muscles were equally affected (22.22%). The sartorius, adductor longus, gracilis, adductor brevis, semitendinosus, and short head of biceps femoris muscles were relatively spared. A distinctive muscle fatty infiltration pattern, the trefoil with single fruit sign at proximal thigh level that is highly specific for a dystrophinopathy [16], was observed on muscle MRI in patients P2, P3, and P5 (Fig. 2D–G).

### Genomic variants identified in routine genetic testing

Multiplex ligation-dependent probe amplification-analysis of *SGCA*, *SGCB*, *SGCD*, *SGCG*, and *DMD* did not detect any possible disease-causing exonic deletions and/or duplications in the 9 enrolled patients. In the 9 patients, the targeted NGS panel detected 9 predicted missense variants in *DMD* and no pathogenic exonic or canonical splice site variants in the other genes. Three of the 9 predicted *DMD* missense variants, including c.5444A > G [15], c.442A > C [25], and c.511G > C [26], were previously reported and the remaining 6 variants were novel (Table 2). Allele frequencies and in silico analyses of the 9 *DMD* variants were summarized in Table 3. However, the previous studies did not perform muscle-derived experiments to determine effects of the c.442A > C and c.511G > C variants on *dystrophin* mRNA and protein [25, 26]. Furthermore, Sanger sequencing trace of the aberrant *DMD* splicing caused by c.5444A > G was not available in the previous study [15]. In the absence of functional studies supportive of a damaging effect of *DMD* missense variants on *dystrophin* mRNA and protein, 3 previously reported *DMD* variants were interpreted as likely pathogenic variants and the remaining 6 predicted *DMD* missense variants were interpreted as variants of uncertain significance according to the ACMG-AMP guidelines (Table 2).

**Table 2:** Summary of genetic data and pathogenicity interpretation of *DMD* variants according to the standard guidelines

Patient number	cDNA variant	Absence of mRNA and protein studies			Evidence derived from mRNA and protein studies							
		Predicted protein variant	Evidence of pathogenicity	Pathogenicity	Confirmed RNA variant	Confirmed protein variant	Confirmed variant type	Exon	Dystrophin domain	Variant frameness	Evidence of pathogenicity	Pathogenicity
P1	c.2380G > C	p.(Glu794Gln)	PM1, PM2, PP3	VUS	r.[2380g > c,2293_2380del,2374_2380del]	p.[Glu794Gln,Ala765Argfs*15,Val792Argfs*15]	Splicing	19	R4	Inframe and frameshift	PVS1, PS3, PM1, PM2, PP3	P
P2	c.4977C > G	p.(Asn1659Lys)	PM1, PM2, PP4	VUS	r.4973_5025del	p.Ser1658Argfs*10	Splicing	35	R12	Frameshift	PVS1, PS3, PM1, PM2, PP4	P
P3	c.5444A > G*	p.(Asp1815Gly)	PS3, PM2, PP3, PP4	LP	r.5444_5448del	p.Asp1815Glufs*2	Splicing	38	R14	Frameshift	PVS1, PS3, PM2, PP3, PP4	P
P4	c.329T > C	p.(Leu110Ser)	PM1, PM2, PP3	VUS	r.329u > c	p.Leu110Ser	Missense	6	ABD:CH1	Inframe	PS3, PM1, PM2, PP3	LP
P5	c.442A > C*	p.(Thr148Pro)	PM1, PM2, PP3, PP4, PP5	LP	r.442a > c	p.Thr148Pro	Missense	6	ABD:CH2	Inframe	PS3, PM1, PM2, PP3, PP4, PP5	P
P6	c.511G > C*	p.(Ala171Pro)	PM1, PM2, PP3, PP5	LP	r.511g > c	p.Ala171Pro	Missense	6	ABD:CH2	Inframe	PS3, PM1, PM2, PP3, PP5	P
P7	c.1649G > C	p.(Arg550Pro)	PM1, PM2, PP3	VUS	r.1649g > c	p.Arg550Pro	Missense	14	R2	Inframe	PS3, PM1, PM2, PP3	LP
P8	c.4760T > C	p.(Leu1587Pro)	PM1, PM2, PP3	VUS	r.4760u > c	p.Leu1587Pro	Missense	34	R12	Inframe	PS3, PM1, PM2, PP3	LP
P9	c.5192T > G	p.(Val1731Gly)	PM1, PM2	VUS	r.5192u > g	p.Val1731Gly	Missense	37	R13	Inframe	PS3, PM1, PM2	LP

^*^Variants have been previously reported [15, 25, 26]. Variants were described in relation to the coding DNA reference sequence NM_004006.2, RNA reference sequence NM_004006.2, and protein reference sequence NP_003997.1. ABD, actin-binding domain; CH1-2, calponin homology; R1-24, spectrin-like repeats; VUS, variant of uncertain significance; LP, likely pathogenic; P, pathogenic; PVS1, very strong evidence; PS, strong evidence; PM, moderate evidence; PP, supporting evidence

**Table 3:** Allele frequencies and in silico analysis of *DMD* variants detected in our patients

Genomic variant	cDNA variant	Allele frequency in gnomAD		MutationTaster	Polyphen-2	SIFT	CADD	GERP + + _RS	SpliceAI			
		All populations	Number of hemizygotes						Acceptor Loss	Donor Loss	Acceptor Gain	Donor Gain
g.32519872C > G	c.2380G > C	Absent	Absent	Disease_causing	Possibly damaging	Deleterious	35	5.35	0	0.84 (0 bp)	0	0.17 (7 bp)
g.32383185G > C	c.4977C > G	Absent	Absent	Disease_causing	Probably damaging	Tolerated	27.8	4.62	0	0.44 (− 48 bp)	0	0.91 (5 bp)
g.32366527T > C	c.5444A > G	Absent	Absent	Disease_causing	Probably damaging	Deleterious	34	5.49	0	0.98 (− 4 bp)	0	1.00 (1 bp)
g.32841440A > G	c.329T > C	Absent	Absent	Disease_causing	Probably damaging	Deleterious	26.8	5.68	0	0	0	0
g.32834673T > G	c.442A > C	Absent	Absent	Disease_causing	Probably damaging	Deleterious	25.4	5.51	0	0	0	0
g.32834604C > G	c.511G > C	Absent	Absent	Disease_causing	Probably damaging	Deleterious	25.4	4.65	0	0	0	0
g.32591917C > G	c.1649G > C	Absent	Absent	Disease_causing	Probably damaging	Deleterious	29.5	5.39	0	0	0	0
g.32398712A > G	c.4760T > C	Absent	Absent	Disease_causing	Probably damaging	Deleterious	27.8	5.36	0	0	0	0
g.32381038A > C	c.5192T > G	Absent	Absent	Disease_causing	Possibly damaging	Deleterious	26.6	5.24	0.08 (37 bp)	0	0	0

The cutoff was set to 2.0 for GERP +  + _RS (smaller scores indicating less conservation). CADD predicts a continuous phred-like score ranging from 1 to 99; higher scores indicate a more deleterious case. We used the phred-like score cutoff of 20 for CADD

### Muscle-derived mRNA studies

Muscle-derived mRNA studies of *SGCA*, *SGCB*, *SGCD*, and *SGCG* did not reveal any aberrant transcripts in the 9 patients, which excluded the existence of atypical splicing variants in *sarcoglycan* genes, including pathogenic synonymous variants, non-canonical splice site variants, and deep-intronic splicing variants. *Dystrophin* mRNA studies revealed only missense transcripts that were consistent with the DNA changes in 6 patients (P4–P9). Muscle-derived *DMD* mRNA studies confirmed that 3 predicted *DMD* missense variants, including the c.2380G > C p.(Glu794Gln), c.4977C > G p.(Asn1659Lys), and c.5444A > G p.(Asp1815Gly) variants, were in fact splicing variants and caused frameshift amino acid changes due to aberrant splicing (Tables 2 and 3).

**Fig. 3:**
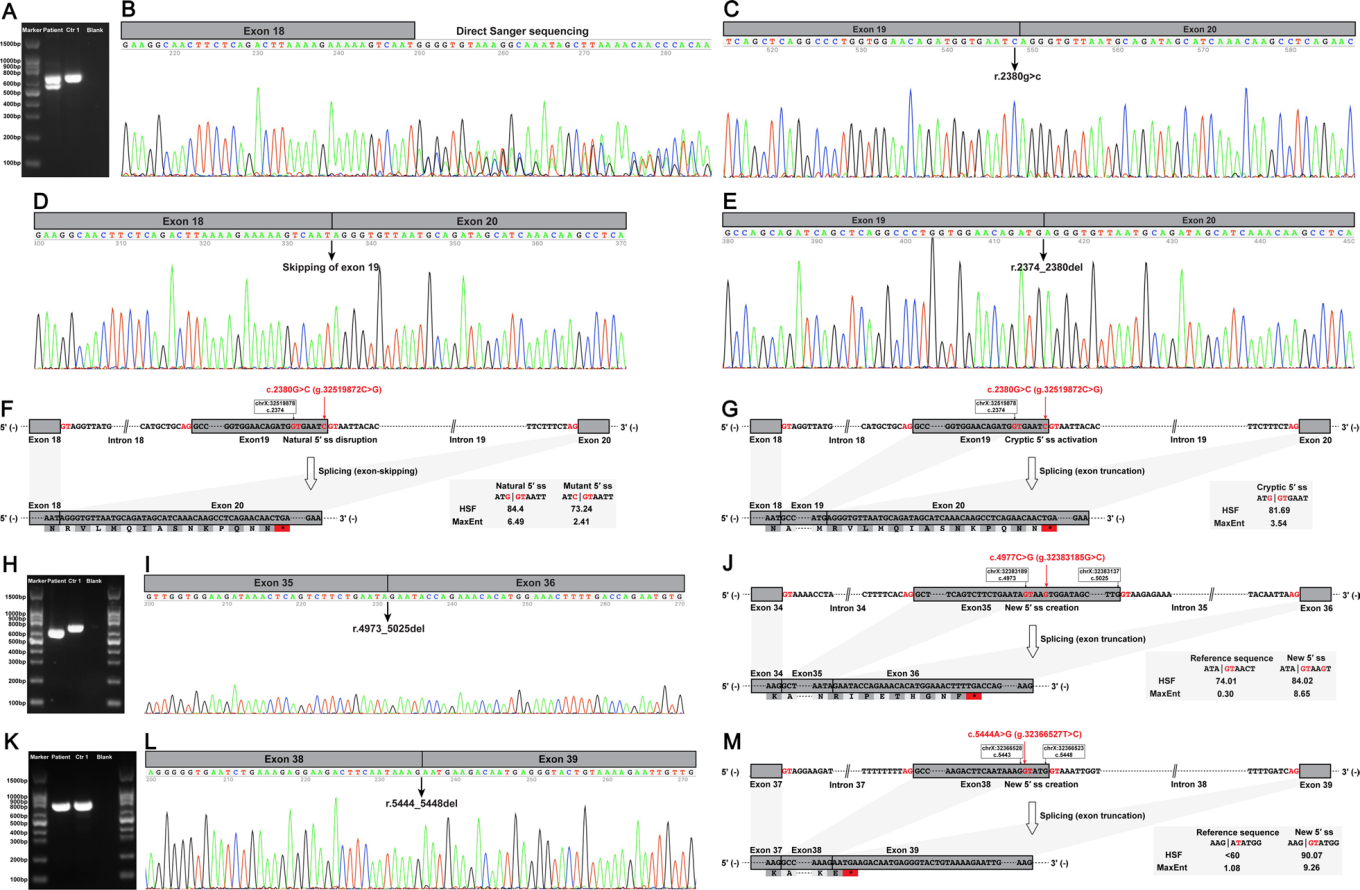
Muscle-derived *DMD* mRNA studies. **A** RT-PCR amplification of the aberrant *DMD* transcripts from patient 1 showed that the lower band was shorter than the expected band, while the upper band was almost the same size as the expected band. **B** Direct Sanger sequencing of the aberrant *DMD* transcripts could not recognize the overlapping sequences. TA cloning of the aberrant *DMD* transcripts revealed three transcripts, including the missense transcript (normal splicing of *DMD* exons 19 to 20; **C**), the skipping of exon 19 (**D**), and a 7 bp deletion of exon 19 (**E**). **F** The schematic of exon 19 skipping caused by the c.2380G > C variant in *DMD*. **G** The schematic of the 7 bp truncation of exon 19 caused by the c.2380G > C variant in *DMD*. **H** RT-PCR amplification of muscle mRNA from patient 2 showed the aberrant *DMD* transcript was shorter than the expected band. **I** Sanger sequencing of the aberrant *DMD* transcript revealed a 53 bp deletion of exon 35. **J** The schematic of the 53 bp truncation of exon 35 caused by the c.4977C > G variant in *DMD*. **K** RT-PCR amplification of muscle mRNA from patient 3 showed the aberrant *DMD* transcript was almost the same size as the expected band. **L** Sanger sequencing of the aberrant *DMD* transcript revealed a 5 bp deletion of exon 38. **M** The schematic of the 5 bp truncation of exon 38 caused by the c.5444A > G variant in *DMD*. The canonical GT–AG splice site pairs at the splice junctions were in red fonts. RT-PCR, reverse transcription-polymerase chain reaction; bp, base pair; *, premature termination codon; 5′ ss, donor splice site; Ctr1, a normal control; Blank, a reagent control; HSF, Human Splicing Finder; MaxEnt, Maximum Entropy

*Dystrophin* mRNA analysis of patient 1 with c.2380G > C revealed three aberrant *DMD* transcripts, including the missense transcript, exon 19 skipping, and a 7 bp truncation of exon 19 (Fig. 3A–E). The c.2380G > C variant disrupted the natural 5′ ss (ATG|GTAATT with an HSF score of 84.4 and a MaxEnt score of 6.49) of exon 19 and activated the cryptic 5′ ss (ATG|GTGAAT with an HSF score of 81.69 and a MaxEnt score of 3.54) in exon 19 (Fig. 3F and G) and thus resulted in the three aberrant splicing events detected in patient 1. SpliceAI also predicted Donor Loss for the natural 5′ ss (0 bp) of exon 19 and Donor Gain for the cryptic 5′ ss (7 bp) in exon 19 caused by c.2380G > C. Both the exon 19 skipping and exon 19 truncation transcripts encoded a frameshift and premature termination codon (PTC), which were targeted for degradation by nonsense-mediated decay (NMD) pathway and caused the severe reduction of dystrophin observed in patient 1 (Fig. 1F and G).

The RT-PCR analysis of *DMD* mRNA in patient 2 with c.4977C > G found a 53 bp truncation of exon 35 (Fig. 3H and I). The c.4977C > G variant created a new 5′ ss (ATA|GTAAGT with an HSF score of 84.02 and a MaxEnt score of 8.65) in exon 35 (Fig. 3J) and caused the aberrant splicing event detected in patient 2. SpliceAI predicted Donor Loss for the natural 5′ ss (− 48 bp) of exon 35 and Donor Gain for the new 5′ ss (5 bp) in exon 35 caused by c.4977C > G. The exon 35 truncation transcript encoded a frameshift and PTC that was degraded by NMD pathway, leading to the absence of dystrophin with isolated revertant fibers observed in patient 2 (Fig. 1J–L).

*Dystrophin* mRNA analysis of patient 3 with c.5444A > G detected a 5 bp truncation of exon 38 (Fig. 3K and L). The c.5444A > G variant created a new 5′ ss (AAG|GTATGG with an HSF score of 90.07 and a MaxEnt score of 9.26) in exon 38 (Fig. 3M) and caused the exon 38 truncation detected in patient 3. SpliceAI predicted Donor Loss for the natural 5′ ss (− 4 bp) of exon 38 and Donor Gain for the new 5′ ss (1 bp) in exon 38 caused by c.5444A > G. The exon 38 truncation transcript encoded a frameshift and PTC targeted by NMD pathway, causing the absence of dystrophin with a few revertant fibers observed in patient 3 (Fig. 1N–P).

### Pathogenicity re-interpretation of detected DMD variants

With evidence derived from mRNA and protein studies, 5 *DMD* variants including 3 previously reported likely pathogenic variants and the remaining 4 *DMD* variants were re-interpreted as pathogenic and likely pathogenic variants, respectively (Table 2). Therefore, 3 patients with *DMD* splicing and frameshift variants (P1–P3) and 6 patients with confirmed *DMD* missense variants (P4–P9) were, respectively, diagnosed with DMD and BMD based on their clinical and pathological characteristics and genetic variants.

## Discussion

The incorrect interpretation of missense variants could cause improper molecular diagnosis and disease management as well as family genetic counselling. However, most *DMD* missense variants are of uncertain significance and their pathogenicity interpretation remains complicated. Herein, we aimed to investigate whether *DMD* missense variants would cause aberrant splicing and re-interpret their pathogenicity based on muscle-derived mRNA and protein studies according to the standard guidelines [23]. Our cases illustrate the challenges in diagnosing patients with uncertain *DMD* missense variants and the significance of muscle biopsy for confirmatory genetic diagnosis.

Genetic diagnosis of dystrophinopathies can be sometimes challenging due to the existence of atypical pathogenic *DMD* variants [2, 4, 27]. Several types of atypical pathogenic *DMD* variants have been identified in dystrophinopathies, including missense and synonymous variants [15, 28], deep-intronic splicing variants [2, 4, 27], and complex structural variants [2, 4, 27], which caused dystrophinopathies by affecting splicing *cis*-elements and thus leading to aberrant splicing of *DMD* pre-mRNA and subsequent dystrophin abnormalities. Aberrant splicing events caused by missense variants have been observed in some other monogenic disease genes, including the *DYSF* [10], *COL4A5* [11], *PKD2* [12], *APC* [13], and *COL4A3* [14] genes. To date, only one atypical *DMD* missense variant inducing aberrant splicing has been reported in a patient with DMD [15], i.e., the c.5444A > G variant initially predicted to cause a missense amino acid change but in fact causing a frameshift change due to aberrant splicing, which was consistent with the finding in our patient (P3 with c.5444A > G).

Aberrant splicing induced by atypical *DMD* missense variants may occur if the predicted *DMD* missense variants alter essential splicing signals and/or splicing regulatory elements. As the largest gene described in the human genome, the *DMD* gene has a high occurrence rate of de novo variants including predicted missense variants [1]. Accordingly, we speculate that there is more than one atypical *DMD* missense variant that can induce aberrant splicing, which is confirmed by our findings in 2 of the 9 enrolled patients. The two patients (P1 and P2), who were suspected of having a diagnosis of DMD based on their clinical and pathological features, had only 2 predicted *DMD* missense variants that were located in spectrin-like repeats of dystrophin. However, *DMD* missense variants located in spectrin-like repeats are usually of uncertain significance and could not cause a severe DMD phenotype [6]. Hence, we performed muscle-derived *DMD* mRNA studies to explore the possible mechanisms underlying the genotype–phenotype discrepancies in the 2 patients. We demonstrated that the 2 predicted *DMD* missense variants (c.2380G > C and c.4977C > G) were in fact frameshift variants at mRNA level due to aberrant splicing and thus caused a severe DMD phenotype, which were re-interpreted as pathogenic variants according to the standard guidelines [23]. The 2 predicted *DMD* missense variants at DNA level were atypical splicing variants at mRNA level. Our cases indicate that in the genomic era, muscle biopsy and subsequent muscle-derived mRNA and protein studies remain important for clinical and genetic interpretation of uncertain *DMD* missense variants, as they can confirm the effects of genomic variants on *DMD* pre-mRNA splicing and dystrophin expression. To our knowledge, our study is the second report of aberrant splicing induced by *DMD* missense variants in dystrophinopathies. As glucocorticoid therapy has significant side effects and is recommended as a standard of care in DMD patients but not in BMD patients [29], the accurate molecular genetic diagnosis of DMD or BMD is very important before initiating glucocorticoid therapy. Patients with *DMD* missense variants are usually associated with a BMD phenotype [7, 8]. However, the possibility that patients with atypical *DMD* missense variants may present a DMD phenotype should be considered, as atypical *DMD* missense variants may in fact be splicing and frameshift variants due to aberrant splicing.

*DMD* missense variants located in non-key functional domains of dystrophin are particularly challenging to interpret [6]. The dystrophin protein (isoform Dp427m) consists of four main functional domains, including an amino-terminal actin-binding domain, a large central rod domain, a cysteine-rich domain, and a carboxyl-terminal domain [30]. DMD patients typically exhibit complete or almost complete deficiency of dystrophin, whereas BMD patients usually show a slight to partial reduction of dystrophin-C regardless of the expression of dystrophin-R and dystrophin-N [4, 31]. The pathogenicity interpretation of *DMD* missense variants becomes more complicated when they are detected in patients with overlapping clinical and pathological characteristics with sarcoglycanopathies. Under this condition, it is difficult to accurately predict the primary genetic defect based on muscle protein immunoanalysis, as a similar reduction of dystrophin and sarcoglycans can be observed in these patients [16]. Therefore, atypical splicing variants in *sarcoglycan* genes that can be easily missed by routine genetic testing should be excluded before establishing a genetic diagnosis of dystrophinopathy in patients with uncertain *DMD* missense variants and overlapping features with sarcoglycanopathies. Six of our 9 enrolled patients (P4–P9), who were suspected of having a diagnosis of BMD or sarcoglycanopathy based on their clinical and pathological features, had only 6 predicted *DMD* missense variants. Muscle-derived studies of *sarcoglycan* genes excluded atypical splicing variants in *sarcoglycan* genes and a molecular diagnosis of sarcoglycanopathy in them. The 6 predicted *DMD* missense variants were re-interpreted as pathogenic or likely pathogenic based on evidence derived from allele frequencies, functional domains, bioinformatic predictions, and *dystrophin* mRNA and protein studies, which confirmed the genetic diagnosis of BMD in the 6 patients. Consistent with the previous studies [8, 32], the 3 confirmed *DMD* missense variants located in ABD1 are also associated with a BMD phenotype in our study. At variance with the previous studies [8, 33], we find that *DMD* missense variants located in spectrin-like repeats (R2, R12, and R13) can be pathogenic and cause a BMD phenotype, which provides valuable information regarding functional domains of dystrophin.

With the recent explosion and advances in bioinformatics and genomics, clinical geneticists commonly turn to bioinformatic predictive algorithms when encountering variants of uncertain significance including missense variants. The bioinformatic predictive algorithms, like MaxEntScan [19], HSF [20], and SpliceAI [21], can be used as tools to predict alterations in essential splicing signals and/or splicing regulatory elements caused by potential splicing variants. However, predictions of the bioinformatic predictive algorithms can sometimes be wrong, like the Acceptor Loss for the natural 3′ ss of *DMD* exon 37 caused by c.5192T > G predicted by SpliceAI, which was not confirmed by our *DMD* mRNA analysis. Furthermore, the predictive algorithms cannot predict the exact sequences of aberrant splicing transcripts caused by potential splicing variants [19, 20]. Hence, it should be noted that the predictions of such predictive algorithms are only predictive, and the accurate effects of potential splicing variants should be confirmed and verified in mRNA and protein studies.

Our study is not without limitations. First, our study enrolled only 9 patients with predicted *DMD* missense variants and is therefore a small sample size research. Second, our study did not perform muscle western blot analysis to quantify the amount of dystrophin protein.

## Conclusion

In conclusion, our study highlights the importance of muscle biopsy and subsequent muscle-derived mRNA and protein studies for uncertain *DMD* missense variants. We show that the effects of *DMD* missense variants cannot be assessed only by predicted amino acid changes but by changes at mRNA and protein levels. Our study is the second report of aberrant splicing induced by *DMD* missense variants in dystrophinopathies, which contributes to the clinical and genetic interpretation of uncertain *DMD* missense variants. In addition, the novel *DMD* variants identified in our study expand the genetic spectrum of dystrophinopathies.

## Data Availability

The authors confirm that the data supporting the findings of this study are available within the article. Raw sequencing data is available from the corresponding authors upon request.

## Abbreviations

DMD: Duchenne muscular dystrophy
BMD: Becker muscular dystrophy
SNVs: Single nucleotide variants
ABD1: Actin binding domain
CK: Creatine kinase
MRI: Magnetic resonance imaging
NGS: Next-generation sequencing
MaxEntScan: Maximum Entropy Scan
HSF: Human Splicing Finder
ss: Splice site
